# Considerations and procedures for acquiring EEG as part of multi-site studies for Rett syndrome and other genetic neurodevelopmental disorders

**DOI:** 10.3389/fnint.2025.1574758

**Published:** 2025-06-09

**Authors:** Joni N. Saby, Eric D. Marsh

**Affiliations:** ^1^Department of Pediatrics, Division of Child Neurology, Children’s Hospital of Philadelphia, Philadelphia, PA, United States; ^2^Department of Neurology and Orphan Disease Center, Perelman School of Medicine, University of Pennsylvania, Philadelphia, PA, United States

**Keywords:** EEG, biomarker, neurodevelopmental disorders, Rett syndrome, evoked potential, CDKL5 deficiency disorder, MECP2 duplication syndrome, FOXG1 syndrome

## Abstract

There is increasing interest in the utility of electrophysiological measures such as resting EEG and evoked potential (EPs) to serve as biomarkers to facilitate therapeutic development for rare genetic neurodevelopmental disorders (NDDs). Research on this topic thus far has been encouraging, but has also revealed the necessity for unique methods when acquiring EEG and EPs in children with genetic NDDs. Details of these methods are typically beyond the scope of research publications, yet are crucial to the quality and ultimately, usability of the data. In the current manuscript, we detail the methods that we have developed for acquiring EEG and EPs as part of multi-site studies with participants with Rett syndrome, CDKL5 deficiency disorder, MECP2 duplication syndrome, and FOXG1 syndrome. By making our methods accessible, we hope to support other groups interested in acquiring EEG and/or EPs as part of clinical trials or research studies with individuals with genetic NDDs, including groups without prior experience with EEG/EP acquisition. The paper is presented as step-by-step procedures followed by a discussion of issues that may arise during acquisition and ways to troubleshoot these issues. We then discuss considerations for choosing EEG equipment and study paradigms and briefly, considerations for data analysis.

## Introduction

EEG abnormalities are common in children with genetic neurodevelopmental disorders (NDDs) including Rett syndrome, MECP2 duplication disorder, CDKL5 deficiency disorder, and Angelman syndrome. Recent studies have demonstrated that many of these abnormalities can be quantified and that quantitative EEG and evoked potential (EP) features correlate with aspects of disorder progression and symptom severity in these groups (Hipp et al., 2021; LeBlanc et al., 2015; Martinez et al., 2023; Roche et al., 2019; Saby et al., 2021; Saby et al., 2022; Saby et al., 2023; Saby et al., 2024; Sysoeva et al., 2020a).

Clinical trials for these and other genetic NDDs are ongoing. These trials face many challenges including the absence of a biomarker(s) of target engagement and treatment efficacy. EEG and EPs have been proposed as candidate measures to fill the need for a biomarker to facilitate therapeutic development for genetic NDDs (Goodspeed et al., 2023; Saby et al., 2020; Sahin et al., 2018). EEG and EPs offer many advantages as biomarkers including being non-invasive, translatable, mobile, and cost-efficient. Furthermore, EEG and EPs are easily repeated and scalable across sites, further underscoring the suitability of these measures for use in longitudinal, multi-site studies, including clinical trials.

Despite these advantages, acquiring EEG and EPs in children with genetic NDDs can be challenging and ensuring data quality with these groups requires unique methods. These methods are typically summarized only briefly in research publications, yet are vital to resulting utility of the data. In light of increasing interest in the utility of EEG and EPs as biomarkers for genetic NDDs, it is timely to discuss these methods, especially considering that many groups that may be involved in the acquisition of these data may not have prior experience with these techniques.

The aim of the current paper is to share methods that we have developed as part of multi-site EEG studies with participants with genetic NDDs. Results of these studies have been published elsewhere (Saby et al., 2021; Saby et al., 2022; Saby et al., 2023; Saby et al., 2024). The focus of this paper is rather on methodology, particularly at the level of data acquisition, to help groups with less experience acquire robust and reproducible EEG and EP data in children with genetic NDDs. The current methods were developed as part of studies of Rett syndrome, MECP2 duplication syndrome, CDKL5 deficiency disorder, and FOXG1 syndrome but these same methods can be similarly applied or modified for studies with other NDDs and developmental and epileptic encephalopathies. In the first half of the paper, we provide a sample standard operating procedure (SOP) including step-by-step instructions for acquiring EEG and EP data in children with genetic NDDs. We then discuss troubleshooting tips for issues that may arise during data acquisition, considerations for equipment and study paradigms, and considerations for data analysis. Given most studies or clinical trials for genetic NDDs will involve multiple sites, special emphasis will be given to multi-site research, although the current methods will similarly apply to single-site studies.

## Overview of procedures

The SOP described below is meant to serve as a guide but readers are encouraged to modify the procedures to fit their particular participant population or research question. For example, the current SOP consists of resting EEG and visual and auditory EPs (VEP/AEP), but groups may choose to acquire only resting EEG and/or EPs. Furthermore, the SOP assumes the use of EEG nets or caps for acquiring the EEG data, but standard electrodes or other systems may alternatively be used. The pros and cons of different EEG setups are discussed later in the manuscript (see *Considerations for equipment and study paradigms*). For most of our studies, we have used high-density, high-impedance Geodesic sensor nets and related equipment (Magstim EGI, Eugene, OR, United States). More detailed procedures for EGI users are provided in Supplementary material.

All of the tasks described in the SOP are passive, without requiring any overt responses from the participant. Therefore, these methods can be used with participants of various ages (infant through adult) and limited motor and communication abilities.

### Environment

Data should be acquired in a cool, dimly lit room free of distractions. This room may be a dedicated lab space or a clinic or other temporary space with a mobile EEG setup. To encourage relaxation and limit electrical noise, overhead lights should be turned off. If the room is too dark without overhead lights, a battery-operated lantern may be useful for achieving optimal luminance. If the data acquisition is taking place in a clinic room or other room with potential distractions, portable partitions, room dividers, or curtains can be helpful to create a “acquisition corner” that separates the participant from the technician and other distractions. It is especially important for the visual task that nothing else in the room may attract attention away from the stimuli.

### Equipment

Each site will need an EEG system (ideally a standardized system across all sites) for acquiring the EEG/EP data. For groups acquiring EPs, each site will also need a stimulus computer and appropriate software program to present the VEP and AEP stimuli and send event triggers to the EEG record. The protocol described below uses E-Prime (Psychology Software Tools, Pittsburgh, PA, United States), to present the VEP and AEP stimuli, but any stimulus presentation software can be used given it integrates with the EEG system to send synchronized trial events. Each site will also need a monitor for presenting the visual stimuli and speakers for presenting the auditory stimuli. A full list of equipment and supplies is provided in Supplementary material.

### Experimental paradigms

All sites should use standardized paradigms for the presentation of the EPs. The current VEP task consists of 400 trials of a reversing black and white checkerboard (0.5 cpd, 2 Hz refresh rate) to elicit a pattern-reversal VEP. The AEP task consists of a sinusoidal 500 Hz tone (300 ms duration) presented for 375 trials with an inter-stimulus interval of 1,000–2,500 ms. For the rationale as well as alternatives to these selected stimuli (see *Considerations for equipment and study paradigms*).

The tasks are presented in the SOP as (1) Resting, (2) VEP, (3) AEP. The order of the VEP and AEP can be reversed but it is recommended to acquire resting EEG first to avoid possible carryover effects from the EP stimuli to the resting EEG. Resting EEG should be recorded for at least 10-15 min to ensure sufficient data is available following segment-based artifact rejection (see *Considerations for data analysis*). Participants may watch a silent movie during the AEP and resting EEG acquisition. Although resting EEG is typically acquired when participants are not engaged in any specific task, a movie is used here in an effort to keep participants calm, still, and awake.

### Data acquisition notes

Throughout the session, the technician should take notes regarding EEG quality as well as the participant’s alertness and/or behavior. It is particularly important to document drowsiness, sleep, and attention to the stimuli as these variables can substantially affect the resulting data and reproducibility between visits. We have found it helpful to have a standardized data acquisition form that technicians fill out at every site for every participant and upload with the EEG files (see Supplementary material).

### Consent and instructions for caregivers

As with any research study or clinical trial, ensure that participants and/or their caregivers have been properly consented prior to initiating any procedures. When confirming EEG visits with families, ask caregivers to wash the participant’s hair the night before (or day of) but to avoid conditioner and other hair products that could interfere with electrode preparation.

## Step-by-step procedures

### Prior to participant arrival

Prior to the participant’s arrival, the technician should set out all materials needed for EEG preparation (EEG nets/caps, measuring tape, syringes, sensory toys, towels, etc.). Arrange and power on the acquisition and stimulus computers, if needed. Once the computers are configured for acquisition, test the VEP and AEP paradigms to ensure the system is working properly and triggers are being received by the EEG system. During the test of the AEP, use a sound level meter (or a sound level meter app) to ensure that the auditory tones are being presented at 65 dB at the approximate location of the participant’s ears (∼60 cm from the speakers). Adjust the sound level on the speakers as necessary.

### EEG preparation

When the participant arrives, allow the participant and caregiver to get comfortable in the room and show them the EEG net or cap. Participants may sit in their wheelchair, on a caregiver’s lap, or independently in a chair during preparation and data acquisition, whichever will be the most comfortable and most likely to discourage movement. Once the participant is comfortable, take appropriate measurements for electrode placement or net/cap size. For children with long hair, pull their hair back in a low ponytail at the base of the head to ease net/cap application.

When ready to place the EEG net/cap, stand directly in front of the child and pull the net/cap down over their head, aligning Cz over the vertex as closely as possible. Keep tension until you have secured the chin strap to avoid the net/cap bunching around the midline. Check for symmetry and adjust as needed. It can be very helpful to have someone else in the room to assist or distract the child with sensory toys during the net/cap placement.

After ensuring the net/cap is properly aligned on the head, prepare the electrodes according to system-specific recommendations and needs (for details and tips for preparing EGI nets, see Supplementary material). Impedances should be within the system recommendations (i.e., < 50 kΩ for high impedance EEG nets; < 10 kΩ for traditional EEG caps and electrodes). In the interest of time and keeping participants from becoming restless, it is ok to move onto acquisition with some channels above recommended impedances if attempts to reduce the impedance are not successful and if EEG preparation has already exceeded 10 min. The exception is the (reference) REF and common or ground (COM/GND) electrodes, which must be under system recommendations to avoid noise in all channels.

For participants with poor neck control, a neck pillow can be extremely helpful for stabilizing the head and reducing head movement and pressure against the outer electrodes during data acquisition. Neck pillows should be placed after the electrodes are prepared and before initiating data acquisition. Coordinators may ask families to bring their own neck pillow to the visit to ensure proper fit and comfort.

### EEG acquisition settings

Acquisition settings must be standardized across all sessions and study sites. Data should be acquired continuously with a predetermined sampling rate (recommended 1,000 or 500 Hz s/s) and without filters to allow for greater flexibility during pre- and post-processing. Each task (resting, VEP, AEP) should be saved as separate files labeled with the participant ID, task, and visit number. Technicians may apply a filter to help visualize the data during acquisition (recommended 1-40 Hz) but ensure this is for visualization only and is not being applied to the raw EEG.

### Resting EEG acquisition

**FIGURE 1:**
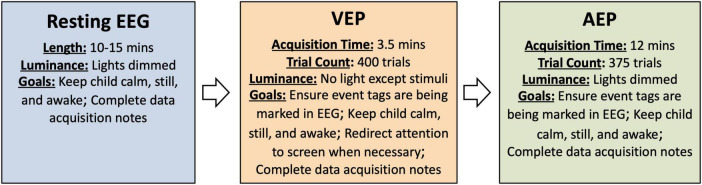
Overview of procedures and goals during acquisition as described in the SOP. Details of the procedures may be modified as discussed in the section on *Paradigm considerations*.

To prepare for data acquisition, re-position the participant, if needed, so that they will be comfortable for resting EEG acquisition. Young children may be most comfortable and likely to remain still on a caregiver’s lap. Older participants may be most comfortable in their wheelchair or sitting in a stable chair, if they are able to sit independently. Once the participant is comfortable and relatively still, begin the recording. EEG should be recorded for 10-15 continuous minutes while participants sit quietly with eyes open. During the EEG acquisition, participants may watch a preferred video or engage in a similarly quiet, passive task such as looking at pictures on an iPad. Lights should be dimmed and the sound from the iPad or other device muted. Throughout the recording, the technician should monitor the participant’s behavior to ensure they are awake with eyes open. Caregivers or technicians may quietly entertain participants by pointing at the screen or playing with toys in an effort to keep participants calm, still, and awake (see Figure 1 for goals during acquisition). At the completion of 15 min, stop the recording, save the file (if not saved automatically), and prepare for the EPs, if applicable. If the participant is becoming restless, the session can be stopped earlier, but aim for a minimum of 10 min of resting EEG.

### VEP acquisition

To prepare for VEP acquisition, use a measuring tape to ensure that the participant is seated 60 cm away from the monitor. Reposition the participant as needed and ensure that they are positioned in the center of the screen. Turn off overhead lights and other significant sources of light in the room (other computer monitors etc.) to encourage attention to the stimuli. Black felt or fabric may also be useful for covering windows in doors and other sources of light if they are significant and distract from the stimuli. Open the provided VEP task in E-prime or alternative stimulus presentation software. Begin the EEG recording and initiate the VEP task. Throughout the VEP, monitor participant’s attention and attempt to re-direct their attention when necessary (see Figure 1). To re-direct attention, the technician or caregiver may try tapping on the screen or shaking an egg-shaker behind the screen to attract participant’s attention to the stimuli. After the completion of the paradigm, stop the recording, save the file (if not saved automatically), and prepare for AEP acquisition, if applicable.

### AEP acquisition

To prepare for AEP acquisition, ensure that the participant is seated 60 cm from the speakers and where the sound was measured at 65 dB during set up. If using two speakers, the speakers should be positioned equidistant from each ear. If using one speaker, the speaker should be placed at the midline to avoid asymmetries in the AEP response. Lights should be dimmed but not completely dark. The participant can watch a movie on a tablet or iPad during the presentation of the auditory stimuli as long as the sound on the movie is muted. Ask others in the room not to talk or create background noise during the AEP acquisition. Open the provided AEP task in E-prime or alternative stimulus presentation software. Begin the EEG recording and initiate the AEP task. During the acquisition of the AEP, participants should be awake with eyes open. Caregivers or experimenters may quietly entertain participants in an effort to keep participants calm, still, and awake (see Figure 1). After the completion of the paradigm, stop the recording and save the file (if not saved automatically).

### Visit completion and data uploads

After all tasks are complete and the participant has left, prepare the files for transfer to the central site or sponsor, if applicable. We have found it useful to compress the raw EEG and EP recordings into a single zipped folder (labeled with participant ID and visit number) prior to transferring. Due to the size of the files, non-compressed files may fail to upload successfully, resulting in a delay in the analysis. If the equipment must be stored after the study visit, power off the amplifier and computers according to the system-provided recommendations before unplugging.

## Troubleshooting

**FIGURE 2:**
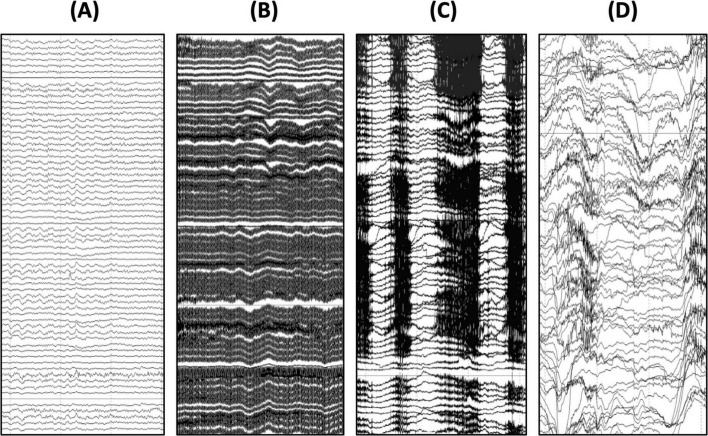
Common artifacts observed in EEG recordings with children with genetic NDDs. **(A)** Example of clean EEG signal contrasted with an EEG signal contaminated by **(B)** 60 Hz electrical artifact, **(C)** teeth grinding artifact, and **(D)** movement artifact. As seen here, these artifacts are much larger in amplitude than the underlying EEG signal and therefore it is important to take precautions to avoid and reduce these artifacts when they arise during data acquisition.

There are a number of technical and behavioral issues that may arise during study visits and negatively impact data quality. Technicians should be familiar with these issues and strategies to help avoid and troubleshoot these issues when then occur (see Figures 2A–D). Below, we discuss some of the more common issues that we have observed in our multi-site studies with children with genetic NDDs.

### 60/50 Hz electrical artefact

Many sites involved in research studies and clinical trials for genetic NDDs may be acquiring data in clinic rooms or other spaces prone to electrical (60/50 Hz) artifact (see Figure 2B). To help reduce the likelihood of 60/50 Hz artifact in the EEG data, unplug non-essential electronics and position the EEG amplifier and the participant away from electrical outlets (at a minimum, 1.5 m away from any outlets). Ensure that the electrode impedances are low (especially the REF and COM/GND electrodes), that the electrodes are contacting the scalp, and the cables are neat and not looped, which can exacerbate line noise. Turn off overhead lights and ensure that the iPad or other device used to present movies during the AEP and resting acquisition is used on battery power only. It is also recommended to use the same testing space routinely so that the team can become familiar with sources of 60/50 Hz artifact in the testing room and ways to mitigate it. For sites with persistent 60/50 Hz artifact in their recordings, a handheld electromagnetic field (EMF) meter can be extremely useful for further troubleshooting and identifying the source(s) of 60/50 Hz artifact. Once identified, try to arrange equipment as far from the source(s) of 60/50 Hz noise as possible. In extreme cases, sites may try acquiring data in a different room to see if the 60/50 Hz artifact is improved.

### Physiological and movement artifacts

Physiological and behavioral factors are another common source of artifacts in EEG from children with genetic NDDs. Artifacts may arise from teeth grinding, breath holding, head shaking, body rocking, and other gross body movements (see Figures 2C,D). These artifacts are substantially larger in magnitude than the underlying brain signals. Therefore, when extensive, these artifacts can significantly limit the usability of the data. The primary way to reduce the occurrence of physiological and movement artifacts is to encourage participants to remain calm and still throughout the session. Presenting a silent movie and dimming the lights are some ways to encourage relaxation. However, some participants will require other means of distraction. For these participants, partner with caregivers to identify ways to encourage the participant to remain still. Choose a movie or show that is familiar and enjoyed by the participant. If the participant is not interested in a silent movie, they may look at photos or games on a phone or tablet or engage quietly with sensory toys. Generally, the potential effects of these activities on the EEG are less concerning than the potential effects of extensive movement or agitation (DiStefano et al., 2019). For some participants, it may also be helpful to offer a weighted blanket during EEG preparation and/or data acquisition. If possible, have a second technician in the room to serve as a behavioral assistant to quietly entertain the participant while the other technician monitors the EEG. If the participant is agitated by the EEG net/cap preparation, offer a break before moving onto data acquisition. Breaks may also be offered in between tasks, as needed. During breaks, the participant may have a snack, play with toys, listen to a song, watch a video with the sound on, or other activities that the caregiver suggests may be calming and distracting. Snacks are generally discouraged during acquisition as this may lead to chewing artifact, but snacks can be a helpful distraction during breaks or during electrode preparation.

It also important to note that all artifacts will be exacerbated by poor electrode preparation. When applying the net/cap, be sure that net/cap size is appropriate and that the electrodes are all making good contact with the scalp. Reduce impedances as much as possible.

### Participants falling asleep

Opposite to the concern of extensive movement and other behavior-based artifacts is the issue of participants falling asleep. Sleep disruptions are common in children with genetic NDDs which may lead to daytime sleepiness. Other factors such as travel to study sites and busy days at the hospital may further contribute to drowsiness and sleep during the EEG session. Although sleep may be permitted and even encouraged for clinical EEGs, the current data should be acquired while participants are awake. To reduce the likelihood of participant falling asleep, avoid scheduling the EEG session during the participant’s typical nap time or at the end of a long day at the hospital. During data acquisition, technicians should monitor participants arousal to ensure they are staying awake. If the participant is falling asleep, the technician or caregiver should attempt to waken the participant by stimulating them and offering a new movie or toy for entertainment. Although most lights should be turned off for the VEP, additional lighting can be used for the AEP and resting acquisition in an effort to keep drowsy participants from falling asleep. It may also be helpful to offer interaction and snack breaks between tasks for participants who appear drowsy. During these breaks, technicians may turn on the overhead lights to further arouse the participant.

### No event triggers

A potential technical issue relevant specifically to the acquisition of the EPs is the possibility of no event triggers. When this occurs, the EP data will not be usable. To avoid this possibility, technicians should test the EP paradigms prior to participant arrival to ensure the EEG acquisition program is receiving event triggers as expected. During data acquisition with the participant, technicians should also check that triggers are being marked in the EEG acquisition program. If event triggers are not being marked in the EEG data, this indicates that the stimulus computer is not communicating with the EEG acquisition software and the task should be aborted and restarted (for troubleshooting details for in Net Station/E-prime users, see Supplementary material).

## Considerations for equipment and study paradigms

### Equipment considerations

The SOP described above can be adapted for use with a variety of different EEG setups. An important caveat when selecting EEG equipment is that all sites should use the same methods to avoid potential site-differences arising from the use of different electrode types and/or EEG systems (Saby et al., 2021). A significant advantage of the Geodesic system (Magstim EGI, Eugene, OR, United States) is that the nets are presoaked in electrolyte allowing for fast preparation time, which can facilitate greater compliance with subsequent study procedures. The nets are also relatively comfortable for the participant as they do not require any paste, gel, or abrasion. To maximize comfort and tolerability of the net, we recommend requesting custom nets without facial electrodes (channels 125-128). Compared to other systems, the EGI system is also relatively user-friendly and easy to learn, even for those without prior EEG experience.

For research studies or clinical trials in which an EGI system is not available at study sites, standard electrodes in combination with clinical EEG systems may be a more feasible option and can be used to acquire the same EEG/EP data (see Saby et al., 2021). Potential drawbacks of using clinical EEG systems are cost (for use of the system and/or EEG technicians) and the need to apply and prepare individual electrodes, which can be time consuming and uncomfortable for the participant. Compared to high-density systems, clinical electrode setups will also have significantly fewer channels, which may deter groups interested in more complex regional and/or localized EEG analyses.

Active electrodes are another option that have been used in studies with children with genetic NDDs (Foxe et al., 2016; Proteau-Lemieux et al., 2022; Sysoeva et al., 2020a). Active electrodes may offer an advantage over other electrode types in that they are less prone to movement artifacts. However, similar to standard electrodes, active electrodes must be prepped with gel prior to acquisition. This process can be time consuming with children with genetic NDDs, which may negatively impact tolerability of subsequent study procedures. Currently, it is not known if any of these EEG setups (EGI, standard electrodes, or active electrodes) offers a clear advantage over others in terms of EEG quality. Therefore, for now, groups may consider availability, cost, and comfort when determining which EEG equipment to use in their study or clinical trial.

### Paradigm considerations

The current protocol consisted of resting EEG, VEP, and AEP. However, existing studies of quantitative EEG in genetic NDDs have indicated that resting measures alone may be useful surrogates of brain function and clinical severity (Hipp et al., 2021; Martinez et al., 2023; Roche et al., 2019; Saby et al., 2022; Saby et al., 2024). Therefore, some groups may choose to only acquire resting EEG, particularly if EP equipment is not available at planned study sites. Similarly, the VEP and AEP protocols may be acquired without resting EEG and/or with modifications to the stimuli used here. For instance, the pattern-reversal (checkboard) VEP may be changed to a flash VEP for participant groups who are unable to maintain fixation during VEP acquisition. Compared to the pattern-reversal VEP, the flash VEP is less dependent on attention, but is also associated with less consistent responses. For the AEP, the 500 Hz tone could be substituted with tone of a different frequency (such as 1,000 Hz). Groups may also desire to shorten the AEP given the current duration of ∼ 12 min is rather long, but we have not explicitly tested the effects of using a shorter paradigm/lower trial count. The AEP could also be modified to include two tones (of different frequencies or durations) to compute a mismatch negativity (MMN) response (see Foxe et al., 2016). One drawback of the MMN task is that it requires a large number of trials and therefore, including MMN may make the protocol too extensive, especially if combined with VEP and resting EEG acquisition. When modifying the AEP protocol, it is important to note that relatively long inter-stimulus intervals (ISIs of > 1,000 ms) may be preferred as longer ISIs result in more consistent and larger responses in some genetic NDDs compared to ISIs less than 1,000 ms (see Kostanian et al., 2023).

## Considerations for data analysis

Analyzing EEG and EP data from individuals with genetic NDDs also requires special considerations that deviate from traditional EEG analyses. Although a detailed review of EEG analysis is beyond the scope of the current paper, we briefly discuss some of these considerations here.

### Dealing with artifacts

Artifacts are to be expected in the EEG data and groups will have to determine how to address them. The most common artifacts are related to muscle movements (e.g., jaw clenching and teething grinding) and body movements (e.g., rocking, swaying, or fidgeting resulting in movement of the net/cap and/or wires from the electrodes to the amplifier; see Figures 2C,D). There are several approaches to dealing with these artifacts in the EEG signal including manual rejection of contaminated segments, automated rejection of contaminated segments, or independent component analysis (ICA). ICA is routinely used in neuroscience research with other populations and offers an advantage over segment rejection in that more data is retained for analysis. Rather than rejecting segments of data, ICA aims to remove only artifact-related components while retaining the background EEG signal. However, ICA methods were developed with the assumptions that artifacts are stereotyped and infrequent, neither of which apply to EEG recordings with children with NDDs. For this reason, most EEG studies with genetic NDDs have used either manual or automated segment rejection rather than ICA (Foxe et al., 2016; Roche et al., 2019; Saby et al., 2023; Saby et al., 2024; Sysoeva et al., 2020b). These approaches result in greater data loss but avoid the possibility of distorting the signal of interest using ICA.

The primary advantage of automatic segment rejection over manual segment rejection is that automatic segment rejection is substantially less time consuming yet yields similar results. The most common criteria for automatic segment rejection in recent EEG studies of genetic NDDs has been signal amplitude either alone or in combination with other features such as line length. Using this approach, segments in which the signal at any channel (or at certain channels of interest) exceeds a predefined threshold (usually between 100 and 250 μV) are automatically rejected. This procedure is generally effective in removing segments with large artifacts related to blinks, movement, and muscle activity, although smaller artifacts may remain. Another disadvantage of this approach is that it can lead to substantial data loss. As an example, in our analyses of resting EEG in 60 girls with Rett syndrome, only 35% of the resting EEG record was retained on average after automated rejection procedures based primarily on signal amplitude (Saby et al., 2024). For most participants, the amount of data retained was still sufficient for analyses, but some participants had to be excluded for insufficient data following automated segment rejection (see *Exclusions*).

### Filter selection

Filtering also warrants special consideration for the pre-processing of resting EEG and EPs from children with genetic NDDs. In traditional EP analysis, minimal high-pass filtering is recommended to avoid distorting the resulting components (Tanner et al., 2015; Zhang et al., 2024). However, individuals with genetic NDDs have abnormally slow background EEGs, which can also distort the averaged waveform and make it challenging to identify components. For this reason, some studies of EPs in participants with genetic NDDs have used an unusually high high-pass filter of 2 or 3 Hz to assist in identifying and analyzing individual components in these populations (Foxe et al., 2016; Saby et al., 2021; Saby et al., 2023; Sysoeva et al., 2020a). For analyses of resting EEG, it is recommended to include these lower frequencies in order to capture abnormalities in delta power. However, it may be necessary to limit analyses of the higher frequencies (> 20 Hz) given the prevalence of muscle artifacts in the EEG from children with genetic NDDs and the potential for these artifacts to contaminate power and other quantitative measures. One conservative approach is to limit all analyses to < 20 Hz (Saby et al., 2024; Sysoeva et al., 2020a). For groups interested in higher frequencies such as high beta and gamma, extra precautions should be taken to ensure that the results are not contaminated by muscle activity.

### Exclusions

An additional consideration relevant to the analysis of both resting EEG and EPs is determining criterion for inclusion in the final analysis. It is likely that some participants may need to be excluded. Reasons for exclusion may be behavioral, such as sleeping during acquisition, or technical, such as no event triggers in the EP files. However, a more common reason for exclusion will be related to excessive EEG artifact resulting in insufficient data for analysis. Groups will have to determine thresholds for inclusion in the analysis based on amount of data retained following artifact rejection, number of accepted trials (for EPs), number of channels rejected/retained, or some combination of these factors. For the EP analyses, additional participants may need to be excluded due to the absence of detectable peaks in the averaged VEP or AEP waveform. Absence of expected peaks has been a common issue in studies of EPs in genetic NDDs, particularly in studies of AEPs (Cutri-French et al., 2020; Kostanian et al., 2023; Saby et al., 2022; Sysoeva et al., 2020a).

The need to exclude participants for these reasons underscores the need for novel, non-traditional methods for analyzing EEG and EPs in participants with genetic NDDs. As described above, ICA has the potential to be a useful technique for removing artifact without losing data, but further research is needed to test and refine ICA and related methods for use with data from children with genetic NDDs (see Auger et al., 2022). Novel approaches such as wavelet thresholding have been proposed as a more conservative alternative to ICA for pediatric EEG and represent another potentially valuable tool for pre-processing EEG data from children with genetic NDDs (Gabard-Durnam et al., 2018; Monachino et al., 2022). For the EPs, novel techniques such as template matching (Miller et al., 2023; Potas et al., 2015) are also needed to reduce data loss and allow for a more inclusive characterization of EPs in these groups.

## Conclusion

The overall goal of the current paper is to support the expansion of research on the utility of electrophysiological measures as biomarkers of brain function and treatment response in children with genetic NDDs. The methods provided here are intended to serve as a guide, particularly for groups interested in this area but unfamiliar with these methods. As with all research methods, our methods are constantly evolving as we learn from new data and new experiences. Therefore, while the current procedures may be used as a foundation, we encourage groups to modify the procedures described here based on new findings and recommendations and to fit their own research and/or clinical goals.

## Data Availability

The original contributions presented in this study are included in this article/Supplementary material, further inquiries can be directed to the corresponding author.

## References

[B1] Auger E. Berry-Kravis E. M. Ethridge L. E. (2022). Independent evaluation of the harvard automated processing pipeline for electroencephalography 1.0 using multi-site EEG data from children with Fragile X Syndrome. *J. Neurosci. Methods* 371:109501. 10.1016/j.jneumeth.2022.109501 35182604 PMC8962770

[B2] Cutri-French C. Armstrong D. Saby J. Gorman C. Lane J. Fu C. (2020). Comparison of core features in four developmental encephalopathies in the rett natural history study. *Ann. Neurol.* 88 396–406. 10.1002/ana.25797 32472944 PMC8882337

[B3] DiStefano C. Dickinson A. Baker E. Jeste S. S. (2019). EEG Data collection in children with ASD: The role of state in data quality and spectral power. *Res. Autism Spectr. Disord.* 57 132–144. 10.1016/j.rasd.2018.10.001 31223334 PMC6585985

[B4] Foxe J. J. Burke K. M. Andrade G. N. Djukic A. Frey H. P. Molholm S. (2016). Automatic cortical representation of auditory pitch changes in Rett syndrome. *J. Neurodev. Disord.* 8:34. 10.1186/s11689-016-9166-5 27594924 PMC5009506

[B5] Gabard-Durnam L. J. Mendez Leal A. S. Wilkinson C. L. Levin A. R. (2018). The harvard automated processing pipeline for electroencephalography (happe): Standardized processing software for developmental and high-artifact data. *Front. Neurosci.* 12:97. 10.3389/fnins.2018.00097 29535597 PMC5835235

[B6] Goodspeed K. Armstrong D. Dolce A. Evans P. Said R. Tsai P. (2023). Electroencephalographic (EEG) biomarkers in genetic neurodevelopmental disorders. *J. Child Neurol.* 38 466–477. 10.1177/08830738231177386 37264615 PMC10644693

[B7] Hipp J. F. Frohlich J. Keute M. Tan W. H. Bird L. M. (2021). Electrophysiological abnormalities in angelman syndrome correlate with symptom severity. *Biol. Psychiatry Glob. Open Sci.* 1 201–209. 10.1016/j.bpsgos.2021.05.003 34841387 PMC8622755

[B8] Kostanian D. Rebreikina A. Voinova V. Sysoeva O. (2023). Effect of presentation rate on auditory processing in Rett syndrome: Event-related potential study. *Mol. Autism* 14:40. 10.1186/s13229-023-00566-1 37885019 PMC10605980

[B9] LeBlanc J. J. DeGregorio G. Centofante E. Vogel-Farley V. K. Barnes K. Kaufmann W. E. (2015). Visual evoked potentials detect cortical processing deficits in Rett syndrome. *Ann. Neurol.* 78 775–786. 10.1002/ana.24513 26332183 PMC7374762

[B10] Martinez L. A. Born H. A. Harris S. Regnier-Golanov A. Grieco J. C. Weeber E. J. (2023). Quantitative EEG analysis in angelman syndrome: Candidate method for assessing therapeutics. *Clin. EEG Neurosci.* 54 203–212. 10.1177/1550059420973095 33203220

[B11] Miller K. J. Müller K. R. Valencia G. O. Huang H. Gregg N. M. Worrell G. A. (2023). Canonical response parameterization: Quantifying the structure of responses to single-pulse intracranial electrical brain stimulation. *PLoS Comput. Biol.* 19:e1011105. 10.1371/journal.pcbi.1011105 37228169 PMC10246848

[B12] Monachino A. D. Lopez K. L. Pierce L. J. Gabard-Durnam L. J. (2022). The HAPPE plus event-related (HAPPE+ER) software: A standardized preprocessing pipeline for event-related potential analyses. *Dev. Cogn. Neurosci.* 57:101140. 10.1016/j.dcn.2022.101140 35926469 PMC9356149

[B13] Potas J. R. de Castro N. G. Maddess T. de Souza M. N. (2015). Waveform similarity analysis: A simple template comparing approach for detecting and quantifying noisy evoked compound action potentials. *PLoS One* 10:e0136992. 10.1371/journal.pone.0136992 26325291 PMC4556619

[B14] Proteau-Lemieux M. Knoth I. S. Agbogba K. Côté V. Barlahan Biag H. M. Thurman A. J. (2022). Corrigendum: EEG signal complexity is reduced during resting-state in fragile X syndrome. *Front. Psychiatry*. 13:867000. 10.3389/fpsyt.2022.867000 35280176 PMC8908967

[B15] Roche K. J. LeBlanc J. J. Levin A. R. O’Leary H. M. Baczewski L. M. Nelson C. A. (2019). Electroencephalographic spectral power as a marker of cortical function and disease severity in girls with Rett syndrome. *J. Neurodev. Disord.* 11:15. 10.1186/s11689-019-9275-z 31362710 PMC6668116

[B16] Saby J. N. Benke T. A. Peters S. U. Standridge S. M. Matsuzaki J. Cutri-French C. (2021). Multisite study of evoked potentials in rett syndrome. *Ann. Neurol.* 89 790–802. 10.1002/ana.26029 33480039 PMC8882338

[B17] Saby J. N. Mulcahey P. J. Benke T. A. Peters S. U. Standridge S. M. Lieberman D. N. (2024). Electroencephalographic correlates of clinical severity in the natural history study of RTT and related disorders. *Ann. Neurol.* 96 175–186. 10.1002/ana.26948 38721759 PMC12045323

[B18] Saby J. N. Mulcahey P. J. Zavez A. E. Peters S. U. Standridge S. M. Swanson L. C. (2022). Electrophysiological biomarkers of brain function in CDKL5 deficiency disorder. *Brain Commun.* 4:fcac197. 10.1093/braincomms/fcac197 35974796 PMC9374482

[B19] Saby J. N. Peters S. U. Benke T. A. Standridge S. M. Swanson L. C. Lieberman D. N. (2023). Comparison of evoked potentials across four related developmental encephalopathies. *J. Neurodev. Disord.* 15:10. 10.1186/s11689-023-09479-9 36870948 PMC9985257

[B20] Saby J. N. Peters S. U. Roberts T. P. L. Nelson C. A. Marsh E. D. (2020). Evoked potentials and EEG analysis in rett syndrome and related developmental encephalopathies: Towards a biomarker for translational research. *Front. Integr. Neurosci.* 14:30. 10.3389/fnint.2020.00030 32547374 PMC7271894

[B21] Sahin M. Jones S. R. Sweeney J. A. Berry-Kravis E. Connors B. W. Ewen J. B. (2018). Discovering translational biomarkers in neurodevelopmental disorders. *Nat. Rev. Drug Discov.* 10.1038/d41573-018-00010-7 [Epub ahead of print]. 30936503 PMC7556736

[B22] Sysoeva O. V. Molholm S. Djukic A. Frey H. P. Foxe J. J. (2020a). Atypical processing of tones and phonemes in Rett Syndrome as biomarkers of disease progression. *Transl. Psychiatry* 10:188. 10.1038/s41398-020-00877-4 32522978 PMC7287060

[B23] Sysoeva O. V. Smirnov K. Stroganova T. A. (2020b). Sensory evoked potentials in patients with Rett syndrome through the lens of animal studies: Systematic review. *Clin. Neurophysiol.* 131 213–224. 10.1016/j.clinph.2019.11.003 31812082

[B24] Tanner D. Morgan-Short K. Luck S. J. (2015). How inappropriate high-pass filters can produce artifactual effects and incorrect conclusions in ERP studies of language and cognition. *Psychophysiology* 52 997–1009. 10.1111/psyp.12437 25903295 PMC4506207

[B25] Zhang G. Garrett D. R. Luck S. J. (2024). Optimal filters for ERP research I: A general approach for selecting filter settings. *Psychophysiology* 61:e14531. 10.1111/psyp.14531 38297978 PMC11096084

