# Supplementary material for: Considerations and procedures for acquiring EEG as part of multi-site studies for Rett syndrome and other genetic neurodevelopmental disorders
Source: Front Integr Neurosci. 2025 Jun 9;19:1574758. doi: 10.3389/fnint.2025.1574758 (PMC12183233; doi:10.3389/fnint.2025.1574758)
Supplement: Supplementary file 1 [file Table_1.docx]

**Supplementary Material**

**Equipment and Supplies List**

**For resting EEG:**

EEG system and associated supplies (nets or electrodes, electrolyte or paste/gel, pipettes, syringes)

Measuring tapes for measuring head circumference and distance from screen/speakers for EPs

iPad or tablet for presenting silent videos

Sensory toys

Battery operated lantern (if needed to obtain dim lighting in room)

Weighted blanket (optional)

Neck pillow (optional and can be provided by family by request)

**For evoked potentials:**

Stimulus computer and stimulus presentation software that integrates with EEG acquisition system

Monitor for presenting VEPs

Speakers for presenting AEPs

Sound level meter for checking sound level

Partitions or room dividers (if needed)

Egg shaker or equivalent to attract participants attention to screen as needed during VEP

**Data Acquisition Form (Sample)**

**General Session Information**

Site/Participant ID

Date of acquisition

Time of acquisition

Initials of technician/experimenter acquiring data

Head Circumference

**Resting EEG**

Was resting EEG recorded? Yes | No

Duration of EEG recording (in minutes)

Luminance level (select one)

☐ Overhead lights on; full-room light

☐ Overhead lights off; dim light from window and/or lantern **Preference for Resting EEG **

☐ No light in room except for monitor

Alertness/State (select one)

☐ asleep

☐ drowsy or closing eyes

☐ awake and mostly still

☐ awake with some movement

☐ awake with persistent movement

☐ awake and distressed/agitated

Session notes (e.g., behavioral difficulties, bad channels, considerable movement, etc.)

**VEP**

Was VEP performed? Yes | No

Was the provided paradigm used? Yes | No

Approximate percentage of time attended to the screen/stimuli (select one)

☐ attended to fewer than 25% of trials

☐ attended to 25% of trials

☐ attended to 50% of trials

☐ attended to 75% of trials

☐ attended to more than 75% of trials

Luminance level (select one)

☐ Overhead lights on; full-room light

☐ Overhead lights off; dim light from window and/or lantern

☐ No light in room except for monitor **Preference for VEP **

Alertness/State (select one)

☐ asleep

☐ drowsy or closing eyes

☐ awake and mostly still

☐ awake with some movement

☐ awake with persistent movement

☐ awake and distressed/agitated

Session notes (e.g., behavioral difficulties, bad channels, considerable movement, etc.)

**AEP**

Was AEP performed? Yes | No

Was the provided paradigm used? Yes | No

Luminance level (select one)

☐ Overhead lights on; full-room light

☐ Overhead lights off; dim light from window and/or lantern **Preference for AEP **

☐ No light in room except for monitor

Alertness/State (select one)

☐ asleep

☐ drowsy or closing eyes

☐ awake and mostly still

☐ awake with some movement

☐ awake with persistent movement

☐ awake and distressed/agitated

Session notes (e.g., behavioral difficulties, bad channels, considerable movement, etc.)

Overall quality of EP/EEG (technologist’s impression, check all that apply)

☐ Limited due to movement or other artifacts

☐ Limited due to sleeping

☐ Limited due to technical reasons

☐ Good quality study

**Detailed Methods for EGI Users**

The same step-by-step procedures provided in the main manuscript are included below but with greater details for users of the EGI EEG system (Magstim EGI, Eugene, OR, USA). The EEG data is recorded using 128-channel Geodesic sensor nets in combination with Net Station Acquisition (Magstim EGI, Eugene, OR, USA). The VEP and AEP stimuli are presented using E-Prime (Psychology Software Tools, Pittsburgh, PA, USA). E-Prime integrates with the EGI EEG system using E-Prime Extensions for Net Station (EENS) to initiate data recording and send synchronized trial events. As with the procedures provided in the main manuscript, groups are encouraged to adapt these procedures to fit their specific participant population or research question.

**Step-by-Step Procedures for EGI Users**

*1. Prior to participant arrival.* Set out all necessary materials and prepare the electrolyte solution using warm water. Ensure the water is warm enough to remain a comfortable temperature until the participant arrives and net is applied. Arrange and power on the acquisition and stimulus computers, if needed, and test the VEP and AEP paradigms to ensure the system is working properly and event triggers are being received by the EEG system. During the test of the AEP, use a sound level meter (or sound level meter app) to ensure that the auditory tones are being presented at 65 dB at the approximate location of the participant’s ears (~60 cm from the speakers). Adjust the sound level on the speakers as necessary.

*2. Applying the net.* When the participant arrives, allow the participant and caregiver to get comfortable in the room and show them the EEG net. Once the participant is comfortable, measure the participant’s head circumference at the largest part of the head to determine the appropriate net size. If a participant is between sizes, choose the smaller net. Soak the net in the prepared electrolyte solution for at least five minutes ensuring that all electrodes are submerged. While the net is soaking, position the participant for net application/data acquisition. Participants may sit in their wheelchair, on a caregiver’s lap, or independently in a chair, whichever will be the most comfortable and most likely to discourage movement during acquisition. For children with long hair, pull their hair back in a low ponytail at the base of the head to ease net application.

Once the net has soaked for at least 5 minutes, remove the net from the electrolyte. When removing the net, some drips may be expected. Avoid towel drying the net as excessive drying may deplete the electrolyte. To apply the net, stand in front of the participant, keep tension on the net with your hands, pull the net straight down over the participant’s head, aligning Cz over the vertex as much as possible. Tighten the chin strap immediately after releasing tension, before making other adjustments. Once positioned, check the net for symmetry and ensure that Cz is positioned over the vertex. Adjust sensors as needed by grabbing and shifting large groups of sensors rather than individual channels. If the net is too far backwards, forwards, or skewed toward one side by more than a few centimeters, it is best to remove the net and try again.

After ensuring the net is properly aligned on the head, quickly go through and check that all sensors are perpendicular to and contacting the scalp. Gently rub each sensor against the scalp to facilitate contact. Connect the net to the amplifier and open Net Station, if it is not already opened. At this point, EEG should be streaming (i.e., streaming set to “on”) but not recording in Net Station. Measure impedances by clicking on the impedance icon. Attempt to reduce the impedance on any channel >50 kΩ by scrubbing the electrode gently against the scalp. If scrubbing is not sufficient to lower the impedance, use a pipette to add a small amount of electrolyte to the electrode. Gently flip the electrode away from the scalp, add a few drops of electrolyte, and then flip it back towards the scalp. Be cautious not to add excess electrolyte as that may lead to “bridging” (when the electrolyte or gel for one electrode connects with the electrolyte or gel of another electrode, resulting in an undesirable bridging of the signals). Attempt to reduce impedances in all channels to < 50 kΩ (indicated by blue shading in Net Station). In the interest of time and keeping participants from becoming restless, it is ok to move onto acquisition with some channels > 50 kΩ if initial attempts to reduce the impedance are not successful and if net preparation has already exceeded 10 minutes. The exception is the REF and COM electrodes, which must be under > 50 kΩ to avoid noise in all channels. Before closing the impedance window, select Save to save impedances for reference during analysis.

For participants with poor neck control, a neck pillow can be extremely helpful for stabilizing the head and reducing head movement and pressure against the outer electrodes. Neck pillows should be placed after the net is prepared and before initiating data acquisition. Coordinators may ask families to bring their own neck pillow to the visit to ensure proper fit and comfort.

*3. EEG acquisition settings.* Hardware settings in Net Station should be set to 1,000 s/s sampling rate. General settings in Net Station should be set to 1 Hz high-pass and 40 Hz low-pass filter (these filters will not impact the raw data but can be helpful for visualizing data during acquisition). Each task (resting, VEP, and AEP) should be saved as a separate file labeled with the participant ID, task, and visit number.

*4. Resting EEG acquisition.* To prepare for data acquisition, re-position the participant, if needed, so that they will be comfortable for resting EEG acquisition. Young children may be most comfortable and likely to remain still on a caregiver’s lap. Older participants may be most comfortable in their wheelchair or sitting in a stable chair, if they are able to sit independently. Once the participant is comfortable and relatively still, initiate the recording in Net Station using the start recording button (indicated by a red circle). EEG should be recorded for 10 – 15 continuous minutes while participants sit quietly with eyes open. During the EEG acquisition, participants may watch a preferred video or engage in a similarly quiet, passive task such as looking at pictures on an iPad. Lights should be dimmed and the sound from the iPad or other device muted. Throughout the recording, the technician should monitor the participant’s behavior to ensure they are awake with eyes open. Caregivers or technicians may quietly entertain participants by pointing at the screen or playing with toys in an effort to keep participants calm, still, and awake (see **Fig. 1** for goals during acquisition). At the completion of 15 minutes, end the recording using the stop recording button in Net Station (indicated by a black square) and prepare for the EPs, if applicable. If the participant is becoming restless, the session can be stopped earlier, but aim for a minimum of 10 minutes of resting EEG.

*5. VEP acquisition.* To prepare for VEP acquisition, use a measuring tape to ensure that the participant is seated 60 cm away from the monitor. Reposition the participant as needed. Turn off overhead lights and other significant sources of light in the room (other computer monitors etc.) to encourage attention to the stimuli. Black felt or fabric may also be useful for covering windows in doors and other sources of light if they are significant and distract from the stimuli. Once ready to begin the task, open the provided VEP task in E-prime. If the E-prime script includes EENS to initiate the recording in Net Station, at this point the EEG data should be streaming (i.e., streaming set to “on”) but not recording. Run the E-prime script and follow the prompts. The E-prime script should now initiate the recording in Net Station and begin to present the VEP stimuli. Check to ensure that event triggers are being received in Net Station. If not, see below for *Troubleshooting Net Station/E-Prime Communications Errors*.

Throughout the VEP, monitor participant’s attention and attempt to re-direct their attention when necessary. To re-direct attention, the technician or caregiver may try tapping on the screen or shaking an egg-shaker behind the screen to attract participant’s attention to the stimuli. If the recording does not automatically stop after the completion of the E-paradigm, manually stop the recording in Net Station using the stop recording button (indicated by a black square).

*6. AEP acquisition.* To prepare for AEP acquisition, ensure that the participant is seated 60 cm from the speakers and where the sound was measured at 65 dB during set up. The lights should be dimmed but not completely dark. The participant can watch a movie on a tablet or iPad during the presentation of the auditory stimuli as long as the sound on the movie is muted. Ask others in the room not to talk or create background noise during AEP acquisition.

Once ready to begin the task, open the provided AEP task in E-prime. If the E-prime script includes EENS to initiate the recording in Net Station, at this point the EEG data should be streaming but not recording. Run the E-prime script and follow the prompts. The E-prime script should now initiate the recording in Net Station and begin to present the AEP stimuli. Check to ensure that event triggers are being received in Net Station. If not, see below for *Troubleshooting Net Station/E-Prime Communications Errors*.

Participants should be awake with eyes open throughout the task. Caregivers or experimenters may quietly entertain participants in an effort to keep participants calm, still, and awake. If the recording does not automatically stop after the completion of the E-paradigm, manually stop the recording in Net Station using the stop recording button (indicated by a black square).

*7. Visit completion and data uploads.* After all tasks are complete and the participant has left, prepare the files for transfer to the central site or sponsor, if applicable. Compress the raw EEG and EP recordings (MFF files) into a single zipped folder labeled with participant ID and visit number and upload this folder to the provided secure server for analysis. Ensure that the files are compressed prior to uploading as non-compressed files may fail to upload successfully. If the equipment must be powered off and stored after the study visit, shut down the amplifier before powering off the computers. To properly shut down the amplifier, select the option in Net Station acquisition to “shut down amplifier and close Net Station” or by opening a Firefox window and selecting shut down amplifier.

*Troubleshooting Net Station/E-Prime Communication Errors for EP acquisition.*  If event triggers are not being received in Net Station or if you receive an error message on the stimulus computer regarding “an error during communication with Net Station”, abort the experiment by selecting ctrl+alt+shift on the E-prime computer. Start a new study in Net Station. Ensure that EEG streaming is set to “on” (but that the EEG is not yet being recorded) prior to initiating the E-prime script. If triggers are still not being received in Net Station (or if you receive another error message in E-prime), you may need to shut down both computers, wait 30 seconds, and then restart both computers.
